# Book Reviews

**Published:** 1870-01

**Authors:** 


### BIBLIOGRAPHICAL NOTICES.

It has been remarked that, as a science and an art, Dentistry is extensive enough to occupy all the time and thought which is not taken up in practical work. This is not so, for it is possible to combine a thorough knowledge of all that pertains to the profession, with a great deal of general knowledge of other subjects.

The Dental practitioner should have such a knowledge of elementary scientific truths beyond his special science, as shall enable him to understand the common physical facts of the world in which he lives, and be capable of appreciating the best thought and knowledge of the times.

The broader a man's culture, the better he can apply such facts as experience may give him, and the sounder will be his conclusions, and the more useful and honorable his career in any profession, to say nothing of the dignity educated members impart to the profession itself.

To those who appreciate the importance of a liberal education, and have a broad and manly view of their profession, and are eager to increase its honor and estimation among men, we recommend the following useful and interesting publications:

*The American Naturalist.* This is an illustrated monthly magazine of Natural History, published by the "Peabody Academy of Science," at Salem Mass., an institution founded by the late George Peabody, and one which will, with many others in this country and Europe, prove a lasting moment of a noble ambition to benefit and instruct mankind. The subscription price of the "Naturalist," is $4.00 a year, and we feel assured that it will prove interesting as well a instructive to every one no difference what his profession may be.

Another useful publication is the *Scientific American*, a well known and very popular weekly journal with all who take special interest in the practical arts and sciences. Every number has sixteen imperial pages, illustrated with engravings of new inventions of every kind. The subscription price is $3.00 a year, Munn & Co., Publishers, 37 Park Row, New York.

*The Manufacturer and Builder*, is another publication similar to the last named, and contains a great deal of useful information upon practical subjects. Its pages are highly embellished with engravings of inventions, designs of buildings and works of art. The subscription price is $1.50 a year, so low that we are at a loss to imagine how such a handsome magazine can be issued on such terms. It is published monthly by Western & Company, 37 Park Row, New York.

Those of our readers living out of the large cities are all, to ome degree at least, interested in agriculture. To such we ould recommend the *American Agriculturist*, published by wrange, Judd & Co., 245 Broadway N. Y., as being one of the best, if not the best paper of the kind issued.

The suggestions of such a publication as this one have proved of great value to those engaged in agricultural pursuits of every nature, such as farming, gardening, fruit growing, &c., &c. Each number of the "Agriculturist" contains from 32 to 44 pages, filled with plain, practical and reliable matter, with many beautiful and instructive engravings. It contains each month a calender of operations to be performed on the farm, orchard, garden, and in the dwelling, with a household department, and also one for children and youth. It has now reached its 29th volume and is issued at $1.50 a year or four copies for $5.

*The Southern Review.* The first number of this Review which is edited by the well known writer Dr. A. T. Bledsoe, assisted by Rev. E. J. Stearns, of Baltimore, Md., was issued in January 1867, and has gained such a reputation that it is now acknowledged to have no superior of its class. It is an organ for Southern men of letters, and among its contributors are to be found the names of some of the ablest writers of the country. All the interesting questions of the day, pertaining to literature, art, science and philosophy are treated in its pages. It is published on the first days of January, April, July and October, at $5.00 per annum.

*The New Eclectic Magazine*, is the title of another valuable Baltimore publication, formed by the consolidation of the "Richmond Eclectic, and "the Land we Love." It is devoted to the publication of selected articles from the best American and Foreign periodicals, and original papers on general literature, science, art, and the educational and material development of the Southern States. It is published by Turnbull & Murdoch, 54 Lexington St., at $4 per annum.

*Once a Month* is the title of an illustrated magazine of good reading for the public, liberally illustrated. It is of high literary excellence, entertaining and instructive. Terms $2 a year, three copies $5 a year. T. S. Arthur & Sons, 809 and 811 Chesnut St. Philadelphia, are the Publishers.

The same Publishers also issue *The Children's Hour*, one of the best magazines for children in existence, at $1.25 a year, or five copies for $5. The well known author T. S. Arthur, edits this work, assisted by a number of popular writers.

*Hitchcock's New Monthly Magazine*, is another publication which is attractive and valuable. It presents a handsome appearance and is finely illustrated, containing also vocal and instrumental music of a high order. The subscription price is $3 per year. Benj. W. Hitchcock, Publisher, 24 Beekman St. N. Y.

One of the oldest magazines, and one too which stands at the head of its class is *Littell's Living Age*, which has now reached its one hundreth volume. It is issued every Saturday, each number containing 64 pages of the valuable literature of the day, embracing reviews, criticisms, tales, poetry, literary, scientific, bibliographical, and historical information. Published weekly at $8 a year. Littell & Gay, 30 Bromfield St., Boston.

### EDITORIAL DEPARTMENT.

